# Comparative Analysis of Prokaryotic Communities Associated with Organic and Conventional Farming Systems

**DOI:** 10.1371/journal.pone.0145072

**Published:** 2015-12-18

**Authors:** Elizaveta Pershina, Jari Valkonen, Päivi Kurki, Ekaterina Ivanova, Evgeny Chirak, Ilia Korvigo, Nykolay Provorov, Evgeny Andronov

**Affiliations:** 1 Laboratory of microbiological monitoring and bioremediation of soils, All-Russia Research Institute for Agricultural Microbiology, Saint-Petersburg, Russia; 2 Saint-Petersburg State University, Saint-Petersburg, Russia; 3 Department of Agricultural Sciences, University of Helsinki, Helsinki, Finland; 4 Natural Resources Institute Finland, Mikkeli, Finland; 5 Laboratory of biology and biochemistry of soils, V.V. Dokuchaev Soil Science Institute, Moscow, Russia; U. S. Salinity Lab, UNITED STATES

## Abstract

One of the most important challenges in agriculture is to determine the effectiveness and environmental impact of certain farming practices. The aim of present study was to determine and compare the taxonomic composition of the microbiomes established in soil following long-term exposure (14 years) to a conventional and organic farming systems (CFS and OFS accordingly). Soil from unclared forest next to the fields was used as a control. The analysis was based on RT-PCR and pyrosequencing of 16S rRNA genes of bacteria and archaea. The number of bacteria was significantly lower in CFS than in OFS and woodland. The highest amount of archaea was detected in woodland, whereas the amounts in CFS and OFS were lower and similar. The most common phyla in the soil microbial communities analyzed were *Proteobacteria* (57.9%), *Acidobacteria* (16.1%), *Actinobacteria* (7.9%), *Verrucomicrobia* (2.0%), *Bacteroidetes* (2.7%) and *Firmicutes* (4.8%). Woodland soil differed from croplands in the taxonomic composition of microbial phyla. Croplands were enriched with *Proteobacteria* (mainly the genus *Pseudomonas*), while *Acidobacteria* were detected almost exclusively in woodland soil. The most pronounced differences between the CFS and OFS microbiomes were found within the genus *Pseudomonas*, which significantly (p<0,05) increased its number in CFS soil compared to OFS. Other differences in microbiomes of cropping systems concerned minor taxa. A higher relative abundance of bacteria belonging to the families *Oxalobacteriaceae*, *Koribacteriaceae*, *Nakamurellaceae* and genera *Ralstonia*, *Paenibacillus* and *Pedobacter* was found in CFS as compared with OFS. On the other hand, microbiomes of OFS were enriched with proteobacteria of the family *Comamonadaceae* (genera *Hylemonella*) and Hyphomicrobiaceae, actinobacteria from the family *Micrococcaceae*, and bacteria of the genera *Geobacter*, *Methylotenera*, *Rhizobium* (mainly *Rhizobium leguminosarum*) and *Clostridium*. Thus, the fields under OFS and CFS did not differ greatly for the composition of the microbiome. These results, which were also confirmed by cluster analysis, indicated that microbial communities in the field soil do not necessarily differ largely between conventional and organic farming systems.

## Introduction

Soil microorganisms can serve as bioindicators of anthropogenic stress experienced by the soil during agricultural use [1]. For a long period of time, biologically valuable soil microorganisms have been studied by isolation and cultivation in laboratory [2]. The next-generation sequencing technologies have intensified exploration of soil microbial diversity and allowed to identify biological indicators, not only among the microbes that can be cultured in vitro, but also among the bacteria and archaea which cannot be cultured [3].

One of the most important challenges of modern agriculture is to determine the effectiveness and environmental impact of systems based on organic or conventional farming (OFS and CFS respectively). Organic farming is considered ecologically friendly and to have less damaging effects on the ecosystem, whereas conventional agriculture is thought to cause significant changes in biocenoses due to the intensive inputs of synthetic fertilizers [4,5,6]. Productivity in CFS is generally higher than in OFS, but the negative impact on the environment associated with the use of a particular type of farming system is debated [7,8,9].

The DOK (short for the German words dynamic, organic and conventional, respectively) experiment is one of the most comprehensive studies on the long-term effects of diverse agricultural techniques on the ecosystem [10]. Studying the soil microbial diversity by pyrosequencing and analysis of the taxonomic markers for bacteria and fungi Hartmann et al. (2014) found that organic fertilizer amendments had a positive effect on the composition of microbial communities and on the α-diversity parameters. Organic matter inputs increased the richness and decreased evenness indices [10]. This effect has been found also in other studies [11,12]. On the other hand, significant increase in richness can lead to positive or neutral effects on evenness in systems with organic fertilizer amendment [13,14,15]. The fluctuations in α-diversity parameters are often explained by predominance of the copiotrophic microorganisms, whose growth is stimulated by organic fertilizers [10,16]. But this statement is equitable only in the short-time experiments, particularly it was shown that copiotrophic bacteria are temporarily stimulated by the addition of organic fertilizers to soil. In the long-run, under stable conditions, the ratio of oligotrophic to copiotrophic bacteria may be greater in OFS than in CFS [17].

Indeed, taxonomic analyses indicate at the phylum level that *Proteobacteria* and *Firmicutes* tend to dominate in the organic farming systems, while *Actinobacteria*, and to a lesser extent *Acidobacteria*, predominate in conventionally managed croplands and natural environments [18,19]. High abundance of the plant growth-promoting bacteria (PGPB), mainly the genera *Rhizobium*, *Bradyrhizobium*, *Mesorhizobium*, *Burkholderia*, *Stenotrophomonas*, *Pseudomonas*, *Sphingomonas* and *Rhodoplanes*, has been documented among the proteobacteria in OFS [13,16,18,2]. Firmicutes in croplands are represented by bacteria capable of degrading various complex organic materials and include, e.g., the genera *Bacillus*, *Clostridium*, *Epulopiscium*, *Paenibacillus* and *Solibacillus* [10]. These data obtained by the modern molecular techniques are partially consistent with the data obtained using bacterial cultivation techniques [20].

Microbial dynamics associated with certain land-use practices must be considered together with the spatial and temporal variations in microbial composition, occurring in soil as a result of plant growth and seasonal changes. Spatial fluctuations in soil microbial communities derive from the unequal distribution of the organic compounds within individual soil aggregates or horizons [21,22,23] and on the different distances from the plant roots [24,25]. As it was shown by van Diepeningen and co-workers the composition of soil microbial community oscillated, depending on the distance remaining from the root. These wavelike patterns were detected both for oligotrophic and copiotrophic bacteria both in OFS and CFS soils but were significantly stronger in conventional croplands [24].

One of the main advantages of employing the pyrosequencing techniques in biodiversity studies is the improvement of knowledge about the impact of agriculture on unculturable microorganisms. The most pronounced effect on soil microbiome revealed in the DOK experiment was the impact of organic fertilizers to the abundance of *Acidobacteria* in soil. *In vitro* cultivation methods for this bacterial phylum are lacking for most of its members, except in rare attempts to define the role of these bacteria in the agricultural systems managed with organic fertilizers amendments [26]. Among acidobacteria, genera *Cand*. *Solibacter* and *Cand*. *Koribacter* have been found exclusively associate with CFS, whereas *Chloracidobacteria* and the RB25 group have been found associated with OFS in previous studies [10].

The aim of this study was to compare long-term impacts of OFS and CFS on microbial diversity in soil. In the experimental station Karila (Mikkeli, Finland) where OFS and CFS have been carried out in adjacent fields for 14 years. The aim was to compare the taxonomic structure of microbiomes in OFS and CFS and to identify microbes specifically inhabiting these ecosystems.

## Materials and Methods

### Soil sampling

Field experiments were carried out under permission of the Natural Resources Institute Finland (formerly MTT AgriFood Research Finland). The field studies did not involve endangered or protected species.

Sampling was done at once from CFS and OFS fields in the experimental station Karila (Mikkeli, Finland) during the season of active plant growth in July 2011. The fields had been cleared from pine-spruce forest in the beginning of 20th century and therefore soil samples were collected from the pine-spruce forest next to the fields included for comparison (Table 1). The soil type was a coarser fine sand in both sampling fields. According to US soil taxonomy soil was sandy Aquic Haplocryod. Soil samples were taken from the top soil layer (10 cm) using soil drill (Ø 1 cm).

**Table 1:** Summary of the cultivation history of the fields sampled in the experimental station “Karila” (Mikkeli, Finland). For details, see S1 Table.

Year	CFS	OFS
**1928**	The forest was cut down	
**1997–2010**	Application of the CFS, regular input of mineral fertilizers	Application of the OFS, regular organic fertilization with cow slurry and green manure
**1997–2006**	Sowing of spring cereals, black currant (in one part of the field)	Crop rotation in 4 steps (1997–2010): 1)spring cereal with ley 2) 3 years of clover-grass ley 3) spring cereal 4)vetch-oats
**2007**	Bare fallow, glyphosate was used	
**2008–2010**	Ley with oats	

At each sampling site three circles (Ø 1 m) were marked and 10 soil subsamples were taken from inside each circle and combined. Hence, three samples (replicates) were obtained for analysis from each type of soil (woodland, CFS and OFS). The distance between the sampling sites was 45 m in average. All samples were immediately transported to the laboratory and stored at -70°C. Coordinates of the sampling sites were the following: woodland soil sample 1 (N61°40'32.46", E27°13'53.70"), 2 (N61°40'32.04", E27°13'55.80") and 3 (N61°40'31.86", E27°13'57.54"); OFS soil sample 1 (N61°40'29.64", E27°13'40.44"), 2 (N61°40'30.00", E27°13'44.40") and 3 (N61°40'30.42", E27°13'48.96"); and CFS soil sample 1 (N61°40'38.22", E27°13'50.04"), 2 (N61°40'37.50", E27°13'51.24") and 3 (N61°40'36.30", E27°13'53.16").

The cultivation history of the fields in Karila is presented in Table 1. Details of the cultivation practices during the last three growing seasons prior to sampling are provided in S1 Table. At the time of sample collection timothy grass (*Phleum pratense*) and meadow fescue (*Festuca pratensis*) were grown as a mixture in both sampled fields (OFS and CFS). Besides analysis of the microbiome, the soil samples from OFS, CFS and woodland were subjected to agrochemical analyses (Table 2).

**Table 2:** Agrochemical properties of soil samples.

	CFS	OFS	WOOD
**Ca (mg/kg)**	1247,33±129,90	1087,00±86,31	242,33±23,13
**P (mg/kg)**	14,70±1,79	10,50±1,22	3,33±0,26
**K (mg/kg)**	135,73±17,25	69,76±8,92	72,70±9,67
**Mg (mg/kg)**	146,67±28,18	117,33±11,21	45,13±5,07
**pH**	6,67±0,09	5,77±0,07	4,43±0,03
**Conductivity**	0,83±0,07	0,63±0,15	0,37±0,03
**Total N**	0,27±0,02	0,34±0,03	0,21±0,01
**Total C**	4,80±0,05	5,49±0,09	5,63±0,12

CFS–conventional farming system, OFS–organic farming system, WOOD–woodland.

### DNA extraction

DNA was extracted from 0.2 g of soil using PowerSoil DNA Isolation Kit (Mobio Laboratories, Solana Beach, CA, USA), which included a bead-beating step, according to the manufacturer’s specifications. Homogenization of the samples was performed using FatsPrep (MP Biomedicals, Santa Ana, CA, USA). The purity and quantity of DNA were tested by electrophoresis in 0.5× TAE buffer on 1% agarose. DNA concentrations were measured at 260 nm using SPECTROStar Nano (BMG LABTECH, Ortenberg, Germany). The average DNA yield was 2–5 μg DNA with the concentration of 10–50 ng/μl.

### Quantitative PCR analyses

Relative abundances of bacterial and fungal small subunit rRNA gene copies were analyzed by quantitative PCR (qPCR) (reaction volume 25 μl) using iQ™ SYBR Green Supermix (BIO RAD, Hercules, USA) and 10 ng of sample DNA. For bacteria, the forward primer Eub338 and reverse primer Eub518 were used [27]. The forward primer arc915 and the reverse primer arc1059r were used for archaea [28]. To estimate bacterial and archaeal small-subunit rRNA gene abundances, standard curves were generated using a 10-fold serial dilution of a plasmid containing a full-length copy of 16S rRNA gene belonging either to the *Escherichia coli* or FG-07 strain of *Halobacterium salinarum* (courtesy of G. Jurgens, University of Helsinki).

All qPCR reactions were run in triplicate. The reaction was carried out in iCycler (BIO RAD, Hercules, USA) using the following: 94°C for 15 min, followed by 40 cycles of 94°C for 30 s, 50°C for 30 s and 72°C for 30 s. Melting curve analyses were done to verify that the amplified products were of the expected size. Fungal and bacterial gene copy numbers were estimated using a regression equation for each assay relating the cycle threshold (Ct) value to the known number of copies in the standards.

Statistical analysis of the qPCR data was carried out using one-way ANOVA in STATISTICA10 Enterprise (www.statsoft.com). Statistical significance was tested by Fischer’s least significant difference (LSD) and Bonferroni adjusted p-values.

### Bar-coded pyrosequencing of bacterial and archaeal communities

The purified DNA templates were amplified with universal multiplex primers F515 5’-GTGCCAGCMGCCGCGGTAA-3’ and R806 5’-GGACTACVSGGGTATCTAAT-3’ [29] targeting the variable region V4 of bacterial and archaeal 16S rRNA genes. Each multiplex primer contained the adapter, 4-bp key (TCAG), 10-bp barcode and primer sequences. The expected length of the amplification product was 400 bp. Purification, pooling and pyrosequencing of the amplicons were performed with reagents according to manufacturer’s instructions (Roche, Branford, USA). Pyrosequencing was carried out using GS Junior system (Roche).

### Bioinformatics of the pyrosequencing-derived dataset

The raw sequences were processed using QIIME ver. 1.8.0 [30]. To reduce sequencing errors, the multiplexed reads were first filtered for quality and grouped according to barcode sequences. Sequences were omitted from the analysis if they were less than 200 bp, had a quality score less than 25, contained uncorrectable barcodes, primers, ambiguous characters or a homopolymer length equal or greater than 8 bp. All non-bacterial ribosomal sequences and chimeras were also removed from the database. In total, 17 311 sequences were obtained with an average of 1923 sequences per library. The dataset was subjected to the normalization procedure resulting in 1100 sequences per sample. The minimum, median and maximum lengths of sequences were 200, 355 and 313 bp, respectively. Similar sequences were clustered into operational taxonomic units (OTUs) with a minimum identity of 97% using *de novo* and *closed reference* algorithms. A representative set of sequences was chosen by selecting the most abundant sequence from each OTU. Representative sequences from each OTU were subjected to RDP naïve Bayesian rRNA Classifier [31] with a confidence level of 80% and aligned using PyNast [32] and Greengenes database [33]. Aligned sequences were used to build a distance matrix with a distance threshold of 0.1 and phylogenetic tree necessary for downstream analysis. Sequence data were archived in SRA database with accession SUB473223.

To compare microbial communities the alpha and beta diversity analyses were performed. To estimate alpha diversity, the indices for richness (observed species, ChaoI) and evenness (PD_whole tree, Shannon evenness, Simpson index) were calculated. The t-test was performed to verify the observed differences. For beta diversity the weighted Unifrac metrics [34] was used to calculate the amount of dissimilarity (distance) between the compared bacterial communities. The results were presented in PCoA analysis using QIIME ver. 1.8.0 [30]. All estimates were measured for the normalized data (normalization was carried out up to the smallest number of sequences present in the sample).

The multiple matrix regression based on Mantel permutations [35] implemented in the phytools R package (http://www.phytools.org) was conducted to reveal the relationships between community composition and different agrochemical properties of soil. To reduce factor space dimensionality (by removing redundant variables) we performed multiple pairwise tests for Spearman rank-order correlation. Significant dependency observed between pH and P allowed us to remove the latter from our feature set.

The abundances of OTUs were compared between samples by calculating the median relative change values for all groups of triplicates. A positive median indicated an increase in abundance, whereas a negative median was taken as evidence for decline of abundance. A basic permutation test was used to infer significance, whereas a jackknife-like resampling approach was applied to test the stability of median estimates.

## Results

### Land use effects on edaphic soil properties

The agrochemical properties of cropland soils managed according to the two different farming systems were rather similar, but differed from the woodland soil despite of the similar soil type (Table 2). The woodland soil had the highest content of organic matter and C/N index, whereas the lowest C/N value was observed in CFS. Soil pH was lowest in the woodland. As for the main biogenic elements, woodland soil was rich in sulfur and manganese, while the croplands were higher in magnesium, calcium and phosphorus (Table 2).

### Relative quantities of bacteria and archaea estimated by qPCR

**Fig 1:**
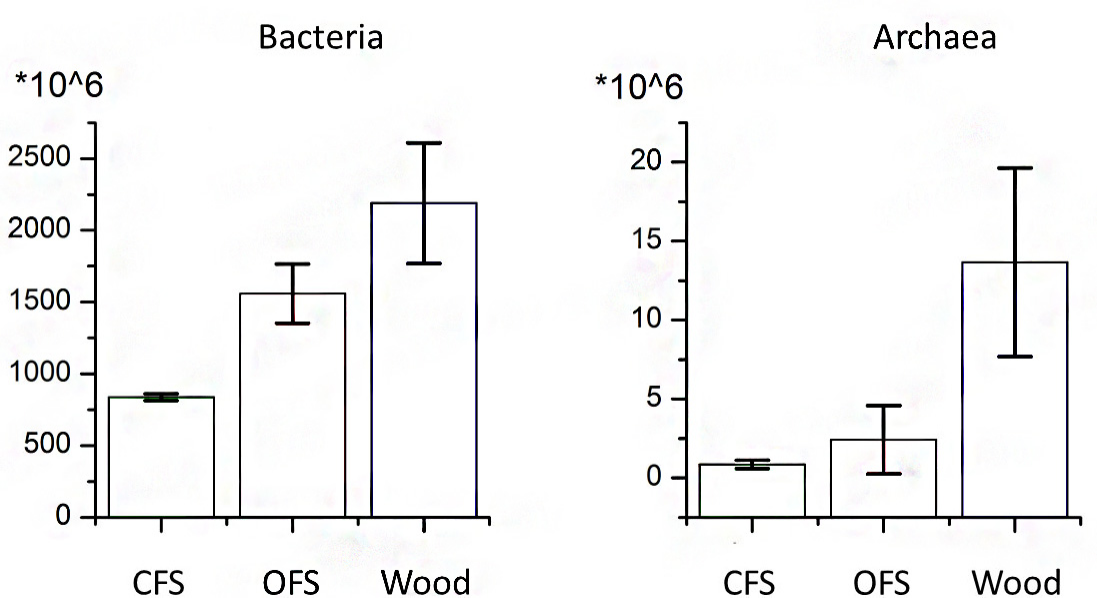
The number of bacteria and archaea per gram of soil, estimated by quantitative PCR. The raw data on the number of 16S rRNA genes per gram of soil, calibrated to the *E*. *coli* and *H*. *salinarum* 16S rDNA copy number, were translated to the number of prokaryotic cells per gram of soil by use of the information on the average number of 16S rRNA copies in bacterial and archaeal genomes deposited in rrnDB database [36]. Error bars indicate standard deviation (n = 3).

The amounts of the bacterial and archaeal biomass estimated by qPCR were expressed as the copy number of rRNA operons per gram of soil and used for comparing the relative abundances of microorganisms in the soil samples. The copy number of ribosomal operons in the genomes of microorganisms varies and is, in average, 4.09 for bacteria and 1.76 for archaea according to the rrnDB database [36]. The experimental data on the average copy numbers of *E*. *coli* and *H*. *salinarum* rRNA operons in soil samples were used to calculate the abundance of bacterial and archaeal communities, respectively. The average number of bacteria in soil was 8.37·10^8^ for CFS, 1.56·10^9^ for OFS and 2.19·10^9^ for woodland (Fig 1). Archaea were about three folds of magnitude less abundant and their average numbers were 8.15·10^5^ for CFS, 2.41·10^6^ for OFS, and 1.37·10^7^ for woodland (Fig 1). These results showed that the population densities of bacteria and archaea were lowest in CFS and highest in woodland (p<0.05). This tendency was particularly noticeable for archaea, whose numbers in the woodland were 2 orders of magnitude higher than in the croplands. The number of bacteria in OFS was significantly higher than in CFS (p < 0.05), whereas the total counts of archaea did not vary between CFS and OFS (Fig 1).

### α-biodiversity of the soil microbial communities

The biodiversity within each individual sample was estimated using richness (number of observed species, Chao1) and evenness (Shannon evenness, Simpson) indices (Table 3).

**Table 3:** Alpha-diversity parameters of soil microbiomes.

Sample ID	Farming system^*a*^		
	CFS	OFS	WOOD
**PD_whole_tree**	30,25±0,87	32,39±0,87	22,24±0,57
**Shannon**	6,58±0,19	6,83±0,19	6,72±0,08
** Simpson**	0,96±0,01	0,97±0,01	0,98±0
** Chao1** ^*b*^	406,53±39,8	450,97±39,8	264,57±9,05
**Number of OTUs**	267,33±11,67	293,67±11,67	218,67±3,71
**Shannon evenness** ^*b*^	0,82±0,02	0,83±0,02	0,86±0,01

^**a**^ CFS, conventional farming system; OFS, organic farming system; WOOD, woodland.

^**b**^The alpha-diversity parameters indicated significant differences (p < 0,05).

Woodland samples had the highest percent of coverage (the number of OTUs to chao1 ratio expressed as a percentage) per library (82.7% in average). The coverage values for OFS and CFS samples were 65.1% and 65.8%, respectively. The observed species richness and Simpson index of dominance were not significantly different between the samples (Table 3).

### Microbial community composition

At the phylum level there were 22 major bacterial taxa present in most of the soils *Proteobacteria* (57.9% in average), *Acidobacteria* (16,1%), *Actinobacteria* (7,9%), *Verrucomicrobia* (2,0%), *Bacteroidetes* (2,7%) and *Firmicutes* (4,8%). The phyla with relative abundance less than 1% were considered rare. They included *Crenarchaeota*, *Armatimonadetes*, BHI80-139, *Chlamydiae*, *Elusimicrobia*, *Fibrobacteres*, GAL15, *Nitrospirae*, TM6, TM7 and WPS-2. Some phyla, such as *Fibrobacteres*, BHI80-139, TM6 and TM7, were found only in croplands. The portion of organisms with unknown taxonomy ranged from 0.6 to 2.1% and was the highest in CFS.

**Fig 2:**
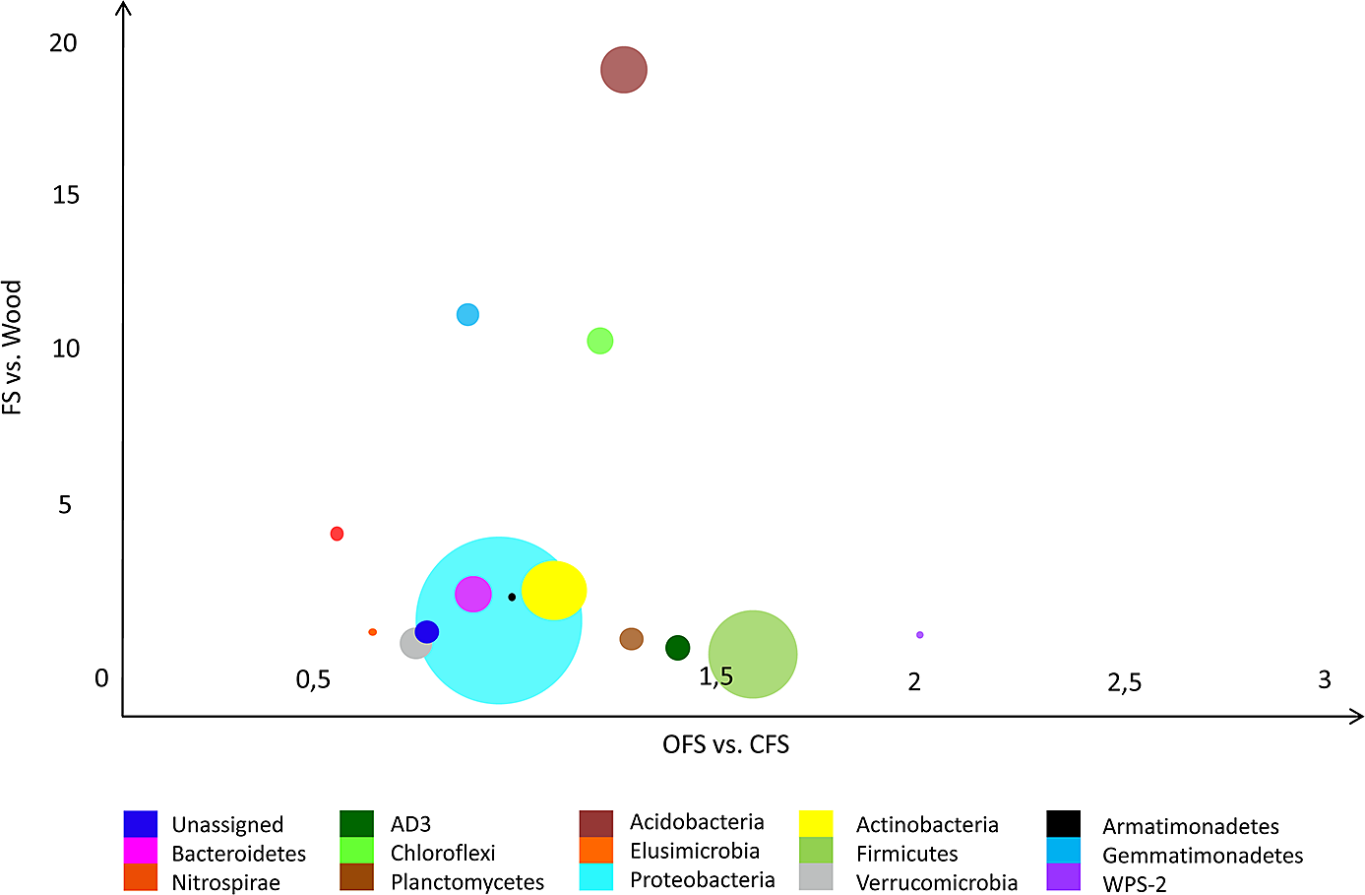
Abundance ratios of the most common bacterial phyla in the soil in organic (OFS) vs. conventional (CFS) farming systems, and the woodland vs. farmland systems (wood vs. FS; FS combines OFS and CFS samples). Circle size indicates the average abundance of the phylum.

At the phylum level, only minor differences were found between the bacterial communities of CFS and OFS, whereas the differences between woodland soil and croplands were more apparent (Fig 2). *Proteobacteria* were among the most abundant phyla in croplands, whereas *Acidobacteria* dominated in the woodland soil (Fig 2). In general, different microbial taxa in woodland soil were more evenly represented, including *Firmicutes*, *Actinobacteria*, *Nitrospira*, *Gemmatimonadetes* and *Chloroflexi*.

**Fig 3:**
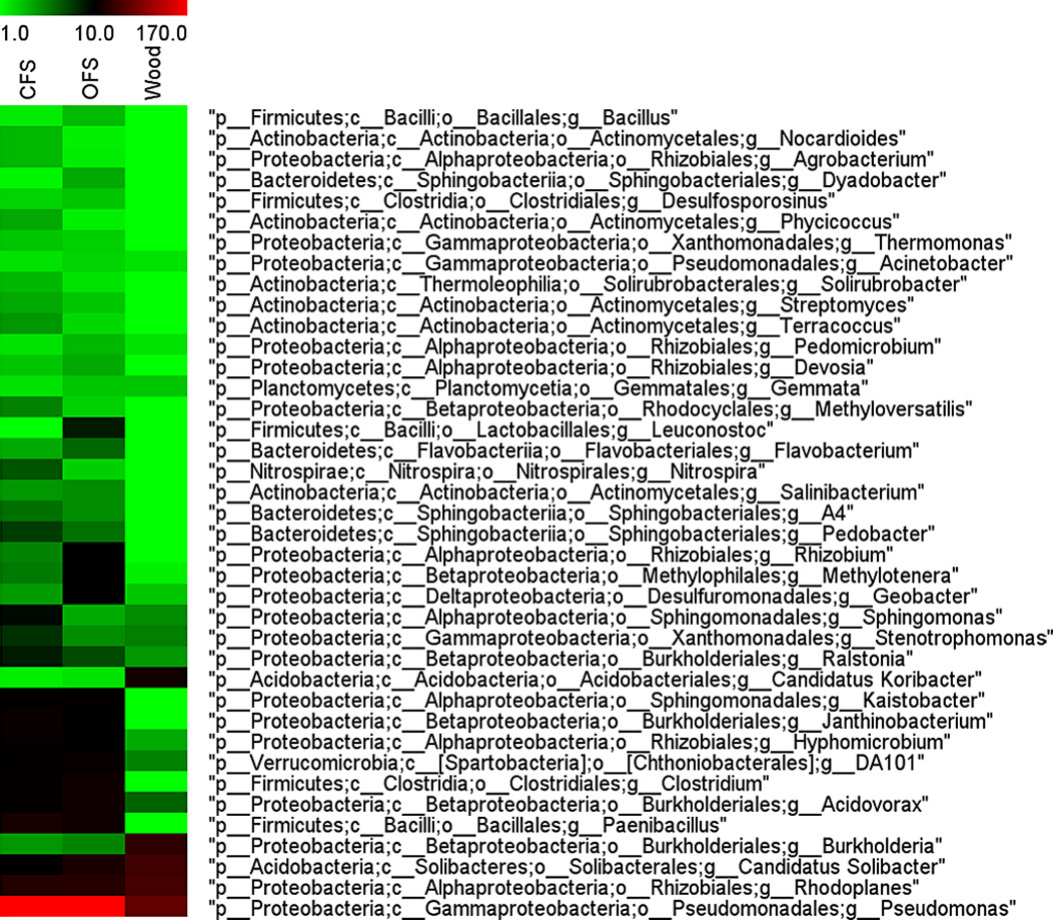
Heatmap comparison of the microbiomes in croplands (CFS and OFS) and the woodland. Colors mark the average relative abundance (in number of sequences per sample) of each bacterial genus within the sample. Only identified genera with total counts exceeding 5 sequences per library are presented.

**Fig 4:**
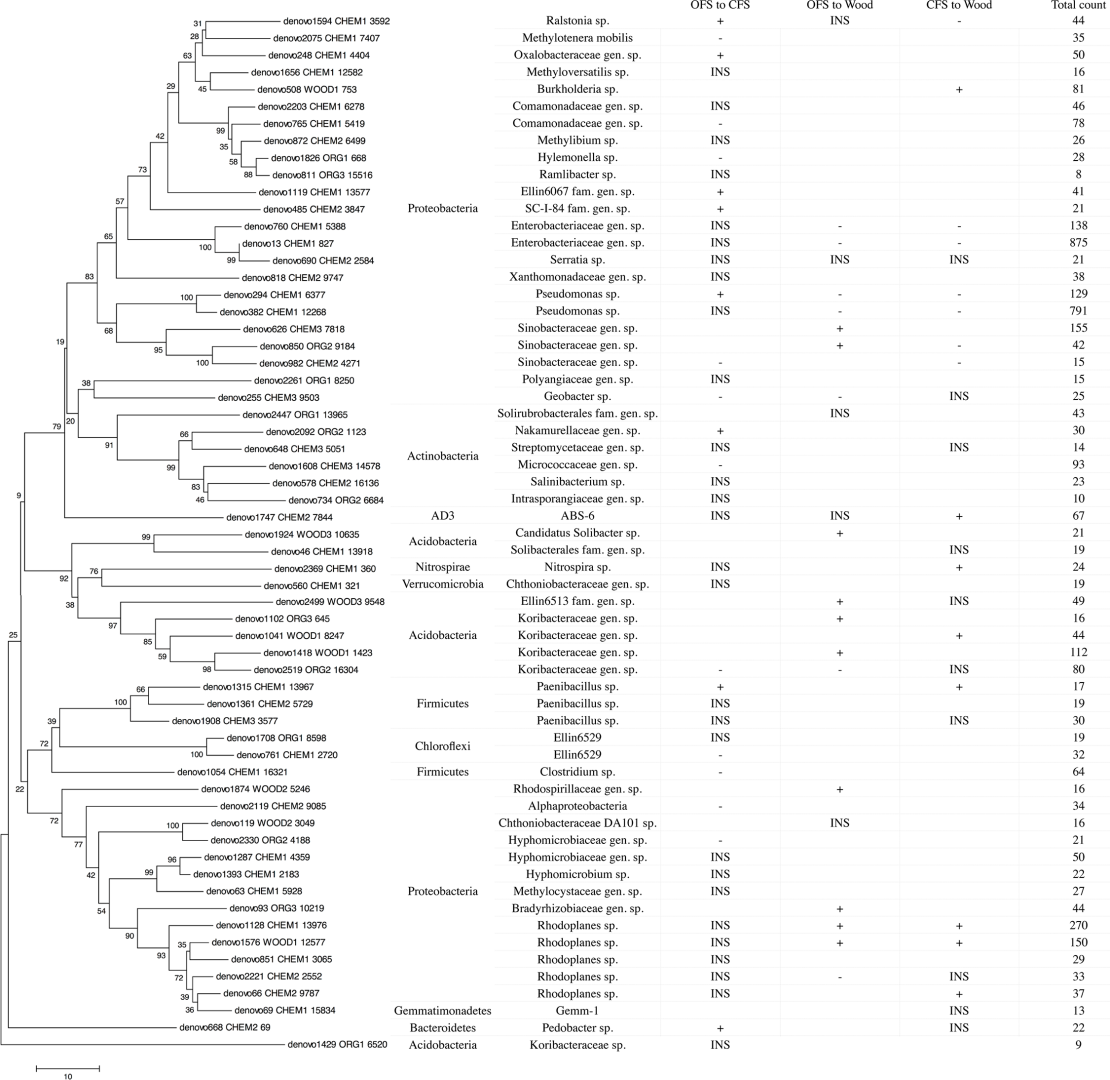
OTUs analyzed in a bootstrapped maximum likelihood phylogenetic tree and their abundance presented in a table. Pairwise tests indicated either an increase (+) or a decrease (–) in abundance between samples of the organic farming system (OFS), the conventional farming system (CFS) and the woodland (Wood). Blank cells indicate insufficient data. The significance of difference was assessed using a permutation test, INS indicates insignificant difference.

The microbiomes of croplands and woodland soils differed markedly in the composition of *Proteobacteria* (Fig 2). Woodland soil was dominated by *Alphaproteobacteria* and *Gammaproteobacteria*, while in croplands *Betaproteobacteria* and *Gammaproteobacteria* were substituting *Alphaproteobacteria*. Bacteria from the families *Pseudomonadaceae* and *Enterobacteriaceae* accounted for more than 80% of the gammaproteobacteria. The family *Pseudomonadaceae* was almost exclusively represented by the genus *Pseudomonas* (Fig 3). The abundance of bacteria of this genus varied between the croplands (16.0% in CFS and 13.2% in OFS). In the woodland soil the number of pseudomonads was only 5.3%. On the other hand, the proteobacterial family *Sinobacteriaceae* counted for more than 7.2% in the woodland soil, as compared with only 1.7% in OFS and CFS. Similarly, betaproteobacteria of the genus *Burkholderia* and alphaproteobacteria of the family *Bradyrhizobiaceae* and the genus *Rhodoplanes* were substantially more common in the woodland soil than in cropland soils (Fig 4). Among the most notable differences in the microbial taxonomic composition between woodland and croplands was the much higher prevalence of the phylum *Acidobacteria* in woodland, in particular, bacteria of the family *Koribacteraceae* (mainly *Candidatus Koribacter*, Figs 3 and 4), the order Ellin6513 and *Solibacterales* (Fig 4).

The main bacterial genera found in soil microbiomes are shown in Fig 3. The most pronounced differences between the CFS and OFS microbiomes were within the genus *Pseudomonas*, which were significantly (p<0,05) more abundant in CFS soil, as compared with OFS. Other statistically significant (p<0,05) differences in the taxonomic composition between CFS and OFS microbiomes were minor and found among the bacterial genera with frequencies rarely exceeding 1% of all taxa. Compared with OFS, CFS had higher relative abundances of the actinobacteria belonging to the family *Nakamurellaceae*, acidobacteria of the family *Koribacteraceae*, proteobacteria of the groups SC-I-84, Ellin6067, *Oxalobacteriaceae* (particularly the genus *Janthinobacterium*) and *Ralstonia* and bacteria, belonging to the genera *Paenibacillus* and *Pedobacter*. Microbial community in OFS was enriched with proteobacteria of the families *Comamonadaceae* (in particular bacteria of the genera *Hylemonella*), *Sinobacteraceae*, *Geobacteraceae* (*Geobacter sp*.) and *Hyphomicrobiaceae*, actinobacteria from the family *Micrococcaceae* and bacteria from the genera *Methylotenera* and *Clostridium* (Fig 4). In OFS samples, the relative densities of the alphaproteobacterial population were found to be increased, which (according to the overall genera composition presented on Fig 3) can be entirely associated with the genus *Rhizobium* (mainly *Rhizobium leguminosarum*).

### β-diversity analysis

The Unifrac distance matrices were tested to determine if land use had a significant effect on the bacterial and archaeal communities. According to the regression analysis (Table 4), unweighted unifrac distances could be quite precisely predicted based on the data on Ca, Mg, pH and total N in the soil (model R squared ~ 0.95, p-level < 0.0005).

**Table 4:** Multiple matrix regression analysis results of the main agrochemical properties of investigated soils.

Intercept	Ca	K	Mg	pH	Conductivity	Total C	Total N
**Coefficients^a^**							
**6,06E-01**	4,34E-05	-5,43E-06	2,07E-04	4,90E-02	2,70E-02	-2,51E-02	9,37E-02
**p-levels^b^**							
**0,00042**	0,02491	0,95206	0,00491	0,0022	0,13100	0,17583	0,04021

^a^Model R squared = 0.9488065.

^b^Model p-level = 0.0004200042.

**Fig 5:**
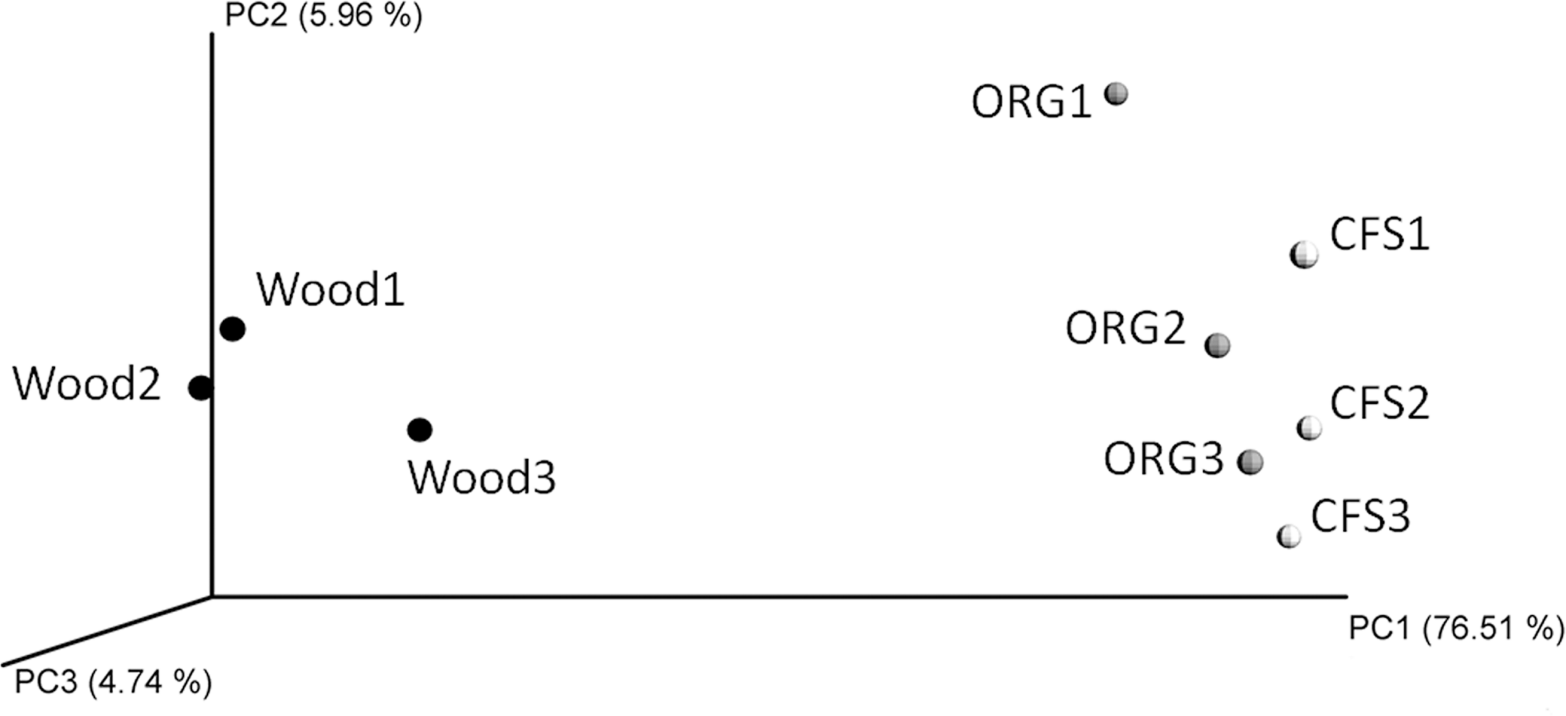
PCoA analysis performed for the weighted unifrac distances of soil microbiomes. Wood, woodland soil; CFS, conventional farming system; OFS, organic farming system. The axes show the percentage of explained variation in unifrac distances.

The community composition analysis as well as the regression results were summarized in PCoA analysis. The croplands formed a separate group clearly separated from the woodland soil (Fig 5). CFS and OFS microbiomes were closer to each other, but there was a trend suggesting a closer relationship of OFS than CFS with woodland soils in the composition of microbiome, as supported by the high percent of explained variation (76,51%) on the corresponding axis.

## Discussion

The highest C/N value and Shannon evenness parameter were observed in the woodland soil as compared with cropland soils, suggesting a more stable microbial community in the woodland than cropland soil. Cropland microbiomes seemed less stable and represented transitional type of the microbial communities, including a few dominant groups [16]. This conclusion is supported by the parameters of α-diversity, which were generally higher in the woodland. The overall community composition, e.g., the presence of physiologically diverse microbiomes in woodland differed from croplands containing mainly copiotrophic bacteria. Thus, regardless of the OFS or CFS practiced, microbiomes in cropland systems were less stable and likely dependent on the external flow of organic matter and macronutrients.

We didn’t find any noticeable differences in α-diversity parameters between OFS and CFS samples, despite of the different farming practices. This result contradicts with the data obtained in a few previous studies reporting on increased richness and decreased evenness parameters of soil microbiomes in OFS [10,11,12].

The results of quantitative PCR revealed a higher total bacterial and archaeal abundance in woodland and OFS soil compared to relatively low amount of microorganisms in CFS soil. These findings may correlate with the changes in the pore-space structure of the soil. Cropland soils usually have big pores with high connectivity, favoring the growth of fungi, whereas woodlands tend to have small isolated pores, creating favorable conditions for development of bacteria [37]. The increased number of bacteria in OFS soil may be also be taken as indication of the inputs of organic fertilizers, which carry not only the various types of organic compounds, but also the indigenous bacteria of manure that may remain in soil for a certain period of time.

Comparison of woodland and cropland soil microbiomes revealed significant differences in the composition of soil bacterial taxa at the phylum level. Woodland microbiome was dominated by acidobacteria including bacteria belonging to the groups *Koribacteraceae*, Ellin6513 and *Solibacteraceae*, considered as oligotrophs in some reports [38]. Increasing numbers of acidobacteria in woodland are likely associated with low pH, as proposed in recent studies [16,19]. Other groups of oligotrophic bacteria were also abundant in woodlands including hemo- and phototrophic bacteria from the groups *Rhodoplanes*, *Nitrospira*, *Rhodospirillaceae* and *Bradyrhizobiaceae* [2]. They are known to fix atmospheric nitrogen, which is an advantage in woodland soil due to the smaller content of available forms of nitrogen, as compared with cropland soils [39]. The presence of oligotrophic bacteria, especially those contributing to autotrophic groups, may indicate high levels of functional diversity leading to diversification of ecological niches. Among other oligotrophic soil bacteria the significant increase in the amount of the genus *Burkholderia* was detected. The representatives of this genus are known to degrade recalcitrant organic matter in soil.

In contrast, the cropland soils were dominated by copiotrophic bacteria from the phyla *Proteobacteria*, including the orders *Pseudomonadales* and *Enterobacteriales*. These bacteria are typical for agroecosystems due to regular mixing of soil and the introduction of nutrients, as well as specific nutritional substrates, such as manure, silage and xenobiotics, whose biodegradability is well described for the *Pseudomonas* group [40]. Predominance of the bacteria belonging to *Pseudomonas* in croplands has been described in several papers using classical microbiological approaches, as well as the new-generation sequencing methods [16,41]. Pseudomonads might be used as the primary bioindicators of the ecological status of the soil due to several reasons: a) they are clearly responding to the changes in the edaphic characteristics of the soil, b) they have high population sizes, and c) they can be detected using *in vitro* cultivation methods and modern molecular methods [16,41,42].

Among the most pronounced differences in the composition of soil microbiomes of croplands soils was the significant increase in the relative amounts of pseudomonads in CFS soils as compared with OFS. Thus, it seems possible to use pseudomonad’s diversity data to distinguish not only woodland and cropland soils but CFS and OFS soils as well. These results are supported by other studies, where it was shown that fluorescent pseudomonads are suppressed in OFS soils [43,44].

Other fluctuations in the certain bacterial groups inhabiting two types of croplands concerned the bacterial genera, whose relative amounts rarely exceeded 1% of the total bacterial counts. Under the conditions of CFS the proportion of bacteria capable of biodegradation of various xenobiotics increased, including the genera *Ralstonia*, *Pseudomonas*, *Paenibacillus* and *Pedobacter*. Another significant finding was the statistically supported increase in the relative abundance of the bacteria from the family *Koribacteriaceae* in CFS compared to OFS, which agrees with results of Hartmann et al. [10]. Thus, we can speculate on the valuable ecological properties of these bacteria in cropland system supplied with mineral fertilizers.

OFS microbiome was dominated by several groups of bacteria, whose appearance in the soil may be caused by the imputes of the spectrum of organic compounds. Particularly this may be concerned as a reason for the increase rates of the bacteria, belonging to the genera *Methylotenera* and *Clostridium*. Methylotrophic bacteria from the genera *Methylotenera* are capable for the utilizing of the methane and its derivatives that accumulate in soil as a result of decomposition of the introduced organic matter [45,46]. Members of the genus *Clostridium* are known to be one of the main anaerobic decomposers of the soil organic matter [47]. The increase in the proportion of clostridia in OFS indicates appearance of anaerobic zones in the soil, which generally form within the soil aggregates [48]. The increase in the proportion of nitrogen-fixing bacteria (*Rhizobium* sp.) and some bacterial genera of the PGPR-group (e.g. the bacteria from the family *Hyphomicrobiaceae*) in OFS soil agrees with previous studies [49,50] and can be explained by the increasing demand for growth factors and mineral elements exhibited by the plant in the absence of mineral fertilizers [51].

The soil microbial communities in cropland soils differed greatly from the microbiome in the soil of the woodland, which was once (ca. 100 years earlier) declared from forest to arable fields. In contrast, the differences between the CFS and OFS microbiomes were much less pronounced, affecting the community composition mainly at the genus level. These conclusions are largely supported by the PCoA analysis, in which OFS and CFS microbiomes are clearly separated from woodland group, whereas the differences between the two cropland systems are minor. Regression analysis showed that the differences observed in microbial composition could be explained by soil chemical properties, among which the soil pH seemed to be the most significant parameter at the phylum level. The amount of biological macroelements, such as Ca and Mg, could be treated in turn as predictors of genus-level variance in the composition of cropland microbiomes. Lower amounts of these mineral nutrients in OFS soil may be one of the reason for the appearance of plant growth promoting bacteria in the corresponding microbiomes, which also agrees with previously reported data [10,13,16].

It is worth mentioning that the data presented in this study are likely dependent on the applied methods of soil sampling, DNA sequencing and bioinformatics analysis. It is well known that soil is highly heterogeneous and sampling may not capture its entire variability. Hence, the results may not be able to explain all the spatial variance in microbiome of the studied habitat. Furthermore, in this study we didn’t investigate the seasonal dynamics of microbial communities, which is likely to exist. Other limitations of the molecular studies of soil microbiomes, such as DNA-extraction and PCR biases [52] also must be considered when interpreting the results of the study.

Keeping the aforementioned limitations in mind, we may summarize the main conclusions as follows. Comparison of the soil microbiomes in two cropping systems (CFS and OFS) with the microbiome in soil of woodland revealed major differences in the agrochemical parameters of soil and the taxonomic composition of microbiomes. Higher C/N ratios and α-diversity parameters as well as the presence of many oligotrophic bacteria in woodland indicate active participation of microorganisms in the deposition of the organic matter increasing its availability for further biodegradation. Croplands represent systems depending partially on the influx of organic or mineral fertilizers, which seems to lead to the predominance of bacteria capable of biodegrading xenobiotics in CFS soils and degrading various organic compounds in OFS soils. Additionally due to the relatively low concentrations of the available mineral macronutrients, OFS soils seem to be dominated by plant growth promoting bacteria. Generally, the use of OFS or CFS had only minor influence on microbial biodiversity in the fields of this study, affecting primarily the genus-level composition of microbiomes. The results provide valuable new information, indicating that carefully managed conventional and organic farming systems may maintain similarly diverse microbial communities, which creates prospects for further research in this area.

## Supporting Information

S1 Table Description of the management history for CFS and OFS experimental croplands during the period from 2007 to 2010. (DOCX)
