# Correction: Comparative Analysis of Prokaryotic Communities Associated with Organic and Conventional Farming Systems

**DOI:** 10.1371/journal.pone.0155155

**Published:** 2016-05-04

**Authors:** Elizaveta V. Pershina, Jari P. T. Valkonen, Päivi Kurki, Ekaterina A. Ivanova, Evgeny L. Chirak, Ilia O. Korvigo, Nykolay A. Provorov, Evgeny E. Andronov

The authors’ middle initials should be included in the author byline. The correct names are: Elizaveta V. Pershina, Jari P.T. Valkonen, Päivi Kurki, Ekaterina A. Ivanova, Evgeny L. Chirak, Ilia O. Korvigo, Nykolay A. Provorov, Evgeny E. Andronov. The correct citation is: Pershina EV, Valkonen JPT, Kurki P, Ivanova EA, Chirak EL, Korvigo IO, et al. (2015) Comparative Analysis of Prokaryotic Communities Associated with Organic and Conventional Farming Systems. PLoS ONE 10(12): e0145072. doi:10.1371/journal.pone.0145072
