# Supplementary material for: Comparative Analysis of Prokaryotic Communities Associated with Organic and Conventional Farming Systems
Source: PLoS One. 2015 Dec 18;10(12):e0145072. doi: 10.1371/journal.pone.0145072 (PMC4684275; doi:10.1371/journal.pone.0145072)
Supplement: S1 Table — (DOCX) [file pone.0145072.s001.docx]

**S1 Table. Description of the management history for CFS and OFS experimental croplands during the period from 2007 to 2010.**

| **Organic farming system (OFS)** | | | | | | | | | |
| --- | --- | --- | --- | --- | --- | --- | --- | --- | --- |
| **Fertlization date** | **Fertilizer** | **Amount of fertilizer** | **Fertilising system** | **Sowing date** | **Crop species** | **Variety** | **Harvest date** | **Harvest system** | **Plant protection** |
| **2007** | | | | | | | | | |
| 11th of May | Cattle slurry | 30 tn/ha | Once in spring | 5th of June | Spring wheat | Kruunu | 3rd of August | Whole crop silage | None |
| 11th of May | Organic trace fertilizer | 200 kg/ha | Once in spring |  |  |  |  |  |  |
| **2008** | | | | | | | | | |
| 9th of May | Cattle slurry | 30 tn/ha | Once in spring | 4th of June | Green manure | Hairy vetch | September | Tilling by rotary cultivator | None |
| **2009** | | | | | | | | | |
| 6th of May | Cattle slurry | 40 tn/ha | Once in spring | 19th of May | Spring wheat + meadow fescue and red clover | Amaretto | 29th of July | Whole crop silage as a nursery for red clover grass | None |
| **2010** | | | | | | | | | |
| 19th of June | Cattle slurry | 30 tn/ha | Once after the first cut | Previous spring | Red clover grass silage | Meadow fescue and red clover | 10th of June 21st of July | Silage 2 cuts/season | None |
| **Conventional farming system (CFS)** | | | | | | | | | |
| **Fertlization**  **date** | **Fertilizer** | **Amount of**  **fertilizer** | **Fertilising**  **system** | **Sowing**  **date** | **Crop**  **species** | **Variety** | **Crop**  **in summer** | **Crop**  **in autumn** | **Plant protection** |
|  |  |  |  |  |  |  |  |  |  |
| **2007** | | | | | | | | | |
| 6th of September | Mineral Y1 | 260 kg/ha | Once in autumn | Late autumn | Autumn wheat Autumn rye | Several for breeders trial | Bare fallow | Autumn cereal | None |
| **2008** | | | | | | | | | |
| None | None | None | None | 26th of May | Green fallow | Timothy and meadow fescue | 29th of July | Cutting and tilling by rotary cultivator | None |
| None | None | None | None | Late autumn | Autumn wheat Autumn rye | Several for breeders trial |  |  | None |
| **2009** | | | | | | | | | |
| None | None | None | None | 25th of May | Green fallow | Timothy, meadow fescue, red clover | 28th of July | Cutting and green mass left in the field | None |
| **2010** | | | | | | | | | |
| None | None | None | None | Previous spring | Green fallow | Timothy, meadow fescue, red clover | 21st of July | Cutting and green mass left in the field | None |

| **µ** | **Soil mechanical fractions %** | | **Content of cattle slurry kg/tn** | | | **Content of Mineral Y1 fertilizer, %** | | **Content of Trace fertilizer, %** | |
| --- | --- | --- | --- | --- | --- | --- | --- | --- | --- |
| 200-2000 | 36 |  | Soluble N | 1.8 |  | N | 26 | N | 0 |
| 60-200 | 40 |  | Total N | 2.9 |  | P | 2 | P | 0 |
| 2-60 | 20 |  | P | 0.51 |  | K | 3 | K | 0 |
| <2 | 4 |  | K | 3.3 |  | S | 2 | Ca | 11 |
|  |  |  | Dry matter | 5.6 % |  | Mg | 0.5 | Mg | 5 |
|  |  |  | Volume | 1000 kg/m3 |  | B | 0.02 | S | 17 |
|  |  |  |  |  |  | Se | 0.0015 | B | 0.3 |
|  |  |  |  |  |  |  |  | Cu | 1 |
|  |  |  |  |  |  |  |  | Fe | 0.3 |
|  |  |  |  |  |  |  |  | Mn | 2 |
|  |  |  |  |  |  |  |  | Mo | 0.05 |
|  |  |  |  |  |  |  |  | Zn | 0.8 |
